# Engaging religious leaders to support HIV prevention and care for gays, bisexual men, and other men who have sex with men in coastal Kenya

**DOI:** 10.1080/09581596.2018.1447647

**Published:** 2018-04-27

**Authors:** Evans Gichuru, Bernadette Kombo, Noni Mumba, Salla Sariola, Eduard J. Sanders, Elise M. van der Elst

**Affiliations:** a Kenya Medical Research Institute – Wellcome Trust Research Programme, Kilifi, Kenya; b Nuffield Department of Population Health, University of Oxford, Oxford, UK; c University of Turku, Turku, Finland; d Department of Global Health, Academic Medical Centre, University of Amsterdam, Amsterdam, The Netherlands; e Nuffield Department of Medicine, University of Oxford, Oxford, UK

**Keywords:** Religious leaders, GBMSM, HIV prevention and care, community engagement, Kenya

## Abstract

In Kenyan communities, religious leaders are important gatekeepers in matters of health and public morality. In a context that is generally homophobic, religious leaders may aggravate or reduce stigmatization of sexual minorities such as gay and bisexual men, and other men who have sex with men (GBMSM). Literature indicates mixed results in efforts to encourage religious leaders to work effectively and sensitively with issues regarding HIV and sexuality. This paper describes the implementation of an engagement intervention with religious leaders from different denominations, which took place following a homophobic hate attack that was led by local religious leaders, at an HIV research clinic for GBMSM on the Kenyan coast. After the homophobic attack, tailored engagement activities, including a comprehensive four-day online sensitivity training course took place between June 2015 and October 2016 in the Kenyan coast. HIV researchers, together with trained GBMSM activists, organized the series of engagement activities for religious leaders which unfolded iteratively, with each subsequent activity informed by the results of the previous one. Facilitated conversations were used to explore differences and disagreements in relation to questions of scripture, mission, HIV, and human sexuality. As a result, researchers noted that many religious leaders, who initially expressed exceedingly negative attitudes towards GBMSM, started to express far more accepting and supportive views of sexuality, sexual identities, and same-sex relations. This paper describes the changes in religious leaders’ discourses relating to GBMSM, and highlights the possibility of using engagement interventions to build trust between research institutes, religious leaders, and GBMSM.

## Introduction

In coastal Kenya, HIV-1 incidence in gay and bisexual men and other men who have sex with men (GBMSM) is high. HIV-1 incidence among men who report sex with men exclusively has been estimated at 35.2 (95% CI 23.8–52.1) per 100 person years. These men have a severalfold higher acquisition risk than men who have sex with both men and women, which has been estimated at 5.8 (95% CI 4.2–7.9) per 100 person years, and 1.1 (95% CI: 0.4–2.8) per 100 person years among heterosexual men followed in a HIV at-risk cohort study in coastal Kenya (Sanders et al., 2013). The Kenyan Ministry of Health and the National AIDS Control Council have both called for the involvement and engagement of various vulnerable populations, including GBMSM, in HIV prevention efforts. However, these efforts are obstructed by laws that criminalize consensual homosexual activity (Cohen et al., 2013), often justified by religious deep-rooted prejudice and patterns of stigmatization against GBMSM (Fay et al., 2011; Mbote, Sandfort, Waweru, & Zapfe, 2016; Nordling, 2014).

Kenya is a deeply religious country; more than 80% of the people adhere to a religion. Protestantism (38%) and Catholicism (28%) are the largest denominations. Islam is practiced by about 11% of the total population, and approximately 85% of those living on the Kenyan coast (Kenya National Bureau of Statistics [KNBS], 2009). Religious leaders play a powerful role in shaping many societal issues (Olson, Cadge, & Harrison, 2006), and often present homosexuality as a threat to Africa’s religious and cultural norms (Epprecht, 2004; Gaudio, 2009; Lornah, 2013; Thoreson & Cook, 2011). Their rejection of same-sex behavior in African society can negatively impact on health research and HIV intervention work involving GBMSM (Endeshaw et al., 2017; Mbote et al., 2016; Watt, Maman, Jacobson, Laiser, & John, 2009), and indeed in 2010, such attitudes led to the closure of the Kenya Medical Research Institute (KEMRI) HIV research clinic in the coast of Kenya for a week (Samura, 2011).

Reacting to rumors that the HIV research clinic was initiating young men to homosexuality and into same-sex marriages, religious leaders led members of the local community to the attack. GBMSM study participants and staff were the main target. The attack was unexpected but not unprecedented – conflicts between communities and medical researchers have taken place across Africa, suggesting local critique of research and a failure on the part of researchers to engage different community stakeholders (Fairhead, Leach, & Small, 2006; Singh & Mills, 2005; Tappan, 2014). Despite the fact that KEMRI had an extensive community engagement program that strengthened relations with those who participated in research (Marsh, Kamuya, Rowa, Gikonyo, & Molyneux, 2008), and HIV researchers adhered to good participatory practice, such as consulting different community stakeholders from the community advisory board, religious leaders were not sufficiently engaged. Subsequent to the attack, specific community engagement staff was hired to work with religious leaders, to explain the nature of HIV prevention and care research with vulnerable populations, and GBMSM in particular (Kombo et al., 2017).

Engagement activities described in this paper, which we call *engagement intervention*, represent an intensive effort to shape discourses of religious leaders with hopes that religious leaders would change their behaviors, views, and attitudes. Evidence of working with religious leaders in HIV prevention has shown positive results. In an inequitable society like Kenya, religious groups care and support sick and vulnerable people, and thus can enhance HIV prevention efforts (Helman, 2007; Kamau, 2009). Downs et al. showed in a cluster-randomized controlled trial, that educating Tanzanian religious leaders about the prevention benefits of male circumcision led to a higher uptake of circumcision in congregations that had, versus congregations that had not, received the intervention (Downs et al., 2017). Similarly, a cluster-randomized trial conducted by Ezeanoule et al. in Nigeria found that encouraging women at church-run baby showers to be tested for HIV had a positive effect on ART uptake and retention (Ezeanolue et al., 2015). A number of studies have also shown that effectively engaging religious leaders can assist with changing negative public views. Willms et al. showed that collaboration with faith-based organizations encouraged heterosexual couples to use a condom (Willms, Arratia, & Makondesa, 2011), while Cornish with female sex workers in India, and Molyneux et al. with GBMSM in Kenya found that engaging religious leaders contributed to decrease of stigmatization of GBMSM, and supported HIV prevention and care for the most vulnerable and affected populations (Cornish, 2006; Molyneux et al., 2016). For religious leaders, however, it is not easy to denounce discrimination on the basis of sexual orientation, (Agadjanian & Sen, 2007; Miller et al., 2011; Williams, Haire, & Nathan, 2017) and so far, very little has been published on how to engage religious leaders in issues regarding homosexuality in particular. This paper seeks to describe the KEMRI research clinic’s engagement intervention and shows that engaging religious leaders can support HIV research and GBMSM access to health care services in Kenya.

## Methods

Drawing on the work of Harro (2000) regarding socialization, stigma, and discrimination, we planned a series of specifically tailored engagement sessions with religious leaders, and members from the GBMSM community in Malindi and environs on the Kenyan coast between June 2015 and October 2016. Facilitated conversations were at the center of the activities. Harro’s conceptual framework, ‘the cycle of socialization’, is closely compatible with our prior cross-cultural work with African health care providers on GBMSM stigma (van der Elst, Gichuru et al., 2013; van der Elst, Gichuru et al., 2015; van der Elst, Kombo et al., 2015; van der Elst, Smith et al., 2013), and the literature on GBMSM stigma (Dijkstra et al., 2015). The model built on a set of concepts, deriving from the introductory guide for health care providers working with GBMSM in Africa (Brown, Duby, Scheibe, & Sanders, 2011), and emphasized the overarching context of stigma and discrimination, linked to religious leaders’ attitudes, offering possible courses of action to ‘choose the direction to change’, interrupt the cycle of socialization and potentially dismantle GBMSM oppression.

Between June 2015 and October 2016, a one-day stakeholders’ meeting, an open day, a three-day workshop, and six comprehensive four-day online sensitivity training courses were organized with local religious leaders from Malindi and nearby areas in the Kenyan coast. Four members of KEMRI’s research team, including two community liaison officers, two HIV behavioral scientists, and six members from coastal GBMSM community-based organizations (CBOs), whose sexual orientation was not shared until the religious leaders had reached a point of being able to receive the information in a non-discriminatory manner, facilitated the engagements. The six GBMSM were trained as facilitators, and had previously assisted with the GBMSM sensitization training for other stakeholders such as health care providers and policy-makers in the Kenyan coast (van der Elst, Gichuru et al., 2015; van der Elst, Kombo et al., 2015).

One hundred thirty-eight out of a total of 141 representatives from six different denominations participated in the engagement activities and included 59 Muslim clerics; 30 Anglican Church pastors; 27 Protestant pastors; 14 Kaya elders (who are considered to be an intrinsic source of ritual power and the origin of cultural identity); four Seventh Day Adventists pastors, and four Catholic priests. All religious leaders were invited on basis of their experiences and visibility in the Malindi community. Three religious leaders refused to participate due to ambiguity regarding the topic.

The engagement activities comprised group work and facilitated conversations, and included an online sensitivity training on GBMSM issues (http://www.marps-africa.org/). This training consisted of four consecutive days of engagement and addressed the following topics: (1) MSM and HIV in sub-Saharan Africa; (2) Stigma; (3) Identity, coming out and disclosure; (4) Anal sex and common sexual practices; (5) HIV and sexually transmitted infections; (6) Mental health, anxiety, depression and substance abuse; (7) HIV prevention measures; and (8) Risk reduction counseling. Twenty-three religious leaders underwent the online training at a time. Modules were designed to be self-completed in 1-2 hours each, and included multiple-choice questions at the end of each module. A score of 71% correct was required to advance to the next module, and upon successful completion of all eight modules, religious leaders were sent a link to download their course certificate. (The learning content of this course is freely available as a web resource and course specifics are documented elsewhere, van der Elst, Smith et al., 2013). Each session was followed by a group discussion, in which religious leaders, and GBMSM and research team members took part.

In each engagement session, facilitators took religious leaders’ perspectives (which were generally constituted of prejudicial attitudes, beliefs and stigmatizing behaviors) as a starting point. They used one or two exercises to initiate conversations, deflect tensions and shift focus if needed. Thereafter, they used carefully facilitated conversations to bring religious leaders’ conduct, thoughts, and feelings out into the open. These conversations were used to review religious leaders’ perspectives of what it means to be human in the society, and what role community and religion play in society at large. Facilitators emphasized the importance of religion in communities, and power that religions and religious leaders wielded. When attitudes of stereotyping, prejudice, and anxiety around homosexuality were uncovered, facilitators applied a carefully crafted approach, and guided the religious leaders through a process of awareness and change within the context of their religious beliefs. For example, facilitators used a commonly shared experience of Kenya’s post-election violence in 2007/2008 to deepen religious leaders’ understanding of the impacts of prejudices, stigma, and public opinion. Facilitated conversations helped religious leaders to develop an understanding of how stigma leads to discrimination, and how they can be better placed to show empathy and support people who face stigma. After this, facilitators guided religious leaders through a discussion about what they could do to ease stigma and discrimination within their religious communities. Exercises, such as ‘turn your habits upside down’ (an exercise on the importance of equity, and how religious leaders can support the advancement of equity for all) challenged and helped leaders to critically appraise own attitudes in the context of their leadership in the community.

Facilitators conducted all sessions in Kiswahili, and religious leaders were aware of and consented to the fact that sessions were documented – anonymously – by trained note-takers. Audio recordings were not considered because of participants’ concerns about confidentiality. Changes in attitudes, views, and knowledge overtime, were documented in multiple sources of evidence, including reports, minutes, notes, and post-sensitization open-ended evaluation questions. Data were manually coded, translated and triangulated by two researchers (EvdE and BK) using Braun and Clarke’s analysis for qualitative data (Braun & Clarke, 2006). An initial coding dictionary was developed through discussions of recurring patterns and themes that emerged from detailed notes and reports generated by the study team (research members, facilitators, and note-takers) immediately after the sessions. This dictionary was periodically updated to reflect nuances and emerging constructs identified during the coding process. An independent (non-coding) research team member (ES) assessed inter-coder reliability to ensure rigor and fidelity to the coding dictionary. Furthermore, data were coded with specific attention to changes in the views of the religious leaders at the start of the engagement as well as later in the process: codes relevant to this paper included themes such as homosexuality is taboo; GBMSM have no place in society; fear and hatred of GBMSM; gap in HIV care and knowledge; shortcoming in engagement with religious leaders; and religious leaders’ role in HIV prevention and care. The final analysis presented in this paper emerged after a series of discussions with all team members and included representative quotes that illustrated key findings.

Participants were compensated 1000 Kenyan shillings (approximately $10.00) for their time and transport costs per day. Reimbursement amounts were determined based on previous studies with stakeholder groups, and approved by local representatives and the IRB. Collection of community engagement data is approved by the Ethical Review Board of the Kenya Medical Research Institute and verbal consent is deemed appropriate for anonymous engagement activities.

## Findings

### Starting point: stigma and insufficient knowledge

At the start of the sessions religious leaders expressed, on the whole, extremely homophobic views. As one Catholic priest, expressing his opinion, said:

The number of men who have homosexual tendencies are increasing and a danger to our cultural values. (Catholic priest, male)

Several Muslim leaders could not suppress their abhorrence of homosexual men. One said:

I hate homosexuals and look down at them as lesser beings … (Muslim cleric, male)

These negative views were justified by conservative readings of religious scriptures. As one Catholic priest argued:

We have all heard of Leviticus, where the Bible straight-up says that homosexual behaviour is an abomination. And yes, it does. It also does say that homosexuals should receive the death penalty. (Catholic priest, male)

Similarly, a Muslim cleric quoted Koranic script Sahih Bukhari 7.72.774,8.82.820:

Turn such people (GBMSM) out of your houses … (Muslim cleric, male)

Seventh Day Adventists similarly argued that ‘gayism should not be endorsed by anyone’ and one (displaying his lack of understanding) asked:

Is there a rehab or seminar that train people to quit [being GBMSM]. (Seventh Day Adventist pastor, male)

A Kaya elder was convinced he could treat homosexuality:

I normally experience some mystical powers, informed by my dreams. The powers (spirits) can help me treat the individual’s [homo] sexuality. (Kaya elder, male)

These negative views towards same-sex sexuality were further justified by religious leaders making references to Kenya’s penal code [Sections 162, 163, and 165], which states: ‘Carnal knowledge against the order of nature is criminalized’. Religious leaders, however, were divided about whether scripture can be above the rule of law. On the one hand, they generally recognized that Kenya’s current constitution also offers protection of civil and human rights which they should also respect. In the sphere of human rights, a Catholic priest cited Luke 10:37, ‘the Good Samaritan’ and implicated that all humans have a right for their needs to be addressed without concern for race or social status. On the other hand, especially religious leaders who had taken part in the KEMRI research clinic attack were of the opinion that their positions made them responsible to eradicate homosexuality in their communities. This meant that they felt they needed to lead processes of social isolation, or ‘pushing out’ of the ‘sinned’ individuals from society.

Negative attitudes were often supported by a lack of sexual education in general and inexperience talking about sexuality in any way. As one clergy from the Anglican Church of Kenya (ACK) shared:

Since my childhood, I have never heard about coital or non-coital sex … (ACK clergy, male)

and another leader from the Anglican Church asked:

How can two men be in love or make love, as sex happens at a place where no reproduction happens … don’t they feel what they do is wrong? (ACK clergy, male)

Although most religious leaders said that their religion had official teaching on human sexuality and that these teachings were described in Holy Scriptures, they strongly felt that religious leaders lacked knowledge about HIV care and prevention. For example, there were myths about the causes of HIV. To paraphrase one of the Kaya leaders, only ‘bewitched or cursed’ people presented with HIV.

Religious leaders were not clear about their role in supporting HIV care for GBMSM. Yet, they said that there were social expectations that they would have knowledge and skills in these areas. A Protestant pastor expressed his bewilderment at this:

Many people don’t feel comfortable dealing with their sons who are gay and also HIV infected yet they expect us [religious leaders] to advise on a way forward … but I don’t know either … (Protestant pastor, male)

Responsibility for lack of knowledge of how to combat HIV among GBMSM was partly laid at the door of researchers and those conducting HIV work in the area. Religious leaders mistrusted researchers working with GBMSM and lacked understanding of what the researchers were doing, and why. This was evident in religious leaders’ accusations that researchers promote homosexuality. One Muslim leader, who confessed to having been a ringleader in the clinic attack, justified his position thus:

The lack of community involvement in these initiatives [working with GBMSM] shows that organizations in Africa are funded by Western countries to spread homosexuality. (Muslim leader, male)

Religious leaders identified lack of clear communication and partnership building as major shortcomings of the researchers, and most religious leaders expressed the desire to be further trained on HIV work and stigma reduction toward GBMSM. As a Protestant pastor said:

Give us information, we have been negligent [and] you kept us ignorant, as community leaders we need to reach many people far and wide … (Protestant pastor, male)

### Changes in religious leaders’ discourses relating to GBMSM

Through the engagement processes religious leaders started to generally acknowledge the broader consequences of stigma, particularly in relation to the HIV epidemic. As perspectives changed, most religious leaders started to use alternative scriptures to justify their new perspectives. For example, one female clergy from the Anglican Church said, citing Genesis 1:27:

We are all created in the image of God, who then knows how God looks like? Why should we judge others with ourselves as the measure? (ACK Reverend, female)

Another clergy cited the story of John (chapter 8 in the Gospel of John in the New Testament of the Christian Bible), saying:

John 8 tells a story of an adulterous woman who was condemned by the community but Jesus brought her close to help her. This should be our role as religious leaders; to treat everyone with love. (ACK clergy, female)

Another colleague interpreted Peter 2:17, indicating that a change in how religious leaders spoke about homosexuality:

‘Honor all people. Love the brotherhood. Fear God. Honour the king’. While the Bible disapproves of homosexual acts, it does not condone hatred of homosexuals or homophobia. Instead, we are directed to love everyone. (ACK clergy, female)

These changes were a surprise to religious leaders themselves. A Muslim sheik said:

I never thought I would sit together with ‘mashoga’ (gay people) … the training has helped me to build a bridge that I can now sit with them. We have learned those people are amongst us; we are ready to support them and give them counsel. (Muslim sheik, male)

Or, as a 56 year old Kaya elder confessed:

Since birth I have never had such a training session; ‘usilolijua usiku wa giza’ (knowledge is power). (Kaya elder, male)

Gaining knowledge about HIV and sexuality *was* particularly empowering for religious leaders and they indicated their recognition that if they changed their attitude towards GBMSM, community members would follow their example. The following quote is of a Seventh Day Adventist pastor:

Thinking back to our discussions, how we treat HIV, how we treat GBMSM, this [knowledge] is an eye opener … (Seventh Day Adventist pastor, male)

The leaders recognized their authority in mitigating tensions in their respective communities. ‘The information we have gotten through the sessions we promise to share it with the wider community and with the interfaith group of Malindi’. They also realized that they have a role to play in facilitating GBMSMs’ social acceptance to advance their access to HIV prevention and care. As expressed by a Muslim Clerk:

At the start of the training we didn’t know whether and how we would accept GBMSM in our community … but as we progressed many of us feel that we are now closer to this [GBMSM] community and we have to take the opportunity to set the example, to minister to them as religious leaders. (Muslim clerk, male)

Others indicated that it was an obligation to share their knowledge with their community members. In doing this, one Protestant leader emphasized Hosea 4:6 in the Christian Old Testament, saying:

‘My people are destroyed for lack of knowledge’ … there is need to train religious leaders to equip them with information so that they can reach out to their congregants. (Protestant pastor, male)

### Approaches for reducing stigma

The aim of the engagement activities was to support the religious leaders to change their views about GBMSM, and to actively work towards the reduction of stigmatization and increased social acceptance of GBMSM in their respective communities. This required the facilitators to tackle the powerful prejudices and areas of discomfort brought forward by the religious leaders. The research team identified the following key approaches for reducing stigma:

#### Generate introspection and self-reflection

Religious leaders themselves indicated they had to overcome their own stigma first before being able to guide their congregation and community members. In order to develop self-awareness, the facilitators asked for testimonies of people within the group who had experienced stigma and discrimination. All group members were challenged to listen, interrogate their fears, and show empathy and compassion. This served to provide a safe environment for the GBMSM testimony-givers. Group members were also encouraged to ask questions of the testimony-givers to gain greater clarity on the issues they had not fully understood. GBMSM testimony-givers spoke professionally and openly about their sexuality, and how their sexuality had influenced their social relations. Through subsequent discussions the religious leaders started to understand that they could use their power to influence and persuade community members to follow their lead in alternative ways, i.e. to focus onto developing an understanding of differences between people, lifestyles and the consequences of prejudice, discrimination, and isolation.

#### Create contact with GBMSM

Having access to GBMSM-members and gay members of KEMRI’s research allowed religious leaders to interact with GBMSM to improve mutual understanding. GBMSM life stories and testimonials were used to generate changes in religious leaders’ negative attitudes and beliefs toward same-sex sexuality. Leaders explicitly expressed the desire to be regularly in contact with people who have in-depth or at least basic knowledge about various aspects of GBMSM. As one Kaya elder said:

To be in contact with people who have been in continuous engagement with the GBMSM would help us to reach out to them. (Kaya elder, male)

This was particularly important when male-to-male sexual HIV transmission was discussed.

#### Provide HIV education

Gaps found in religious leaders’ HIV prevention and care knowledge needed to be filled urgently. During online training, brainstorming sessions were used to generate discussion about any issues that came to mind. Small lectures provided conceptual detail, and small group work was used to stimulate participation. Provision of facts, distinguishing myth and gossip from research and truth overcame judgment. Biomedicine was called upon as an expert opinion. This approach, especially when making reference to biological facts, proved a powerful way of reducing stigma. The focus was on improving religious leaders’ understanding of why GBMSM are targeted for HIV prevention and care through exposing the leaders to comprehensive knowledge on sexual biology, HIV/AIDS transmission, protection, and treatment. Religious leaders were also provided with knowledge of how to respond as leaders in HIV-related issues. For this, participants brainstormed about ways they could counsel their congregations on HIV. For example, facilitators asked them to devise response strategies for what they would do if a GBMSM parishioner came asking questions about HIV, or if a GBMSM parishioner came saying he had been diagnosed with HIV.

#### Emphasize responsibility

Sessions placed emphasis on religious leaders’ role in supporting HIV prevention and care for GBMSM, setting them at the forefront of the battle against HIV. The importance of religion in communities was acknowledged, and that this provided an opportunity for them to enhance GBMSM’s access to HIV prevention and care was emphasized. This happened through discussing religious leaders’ counseling role, and the leaders were challenged to acknowledge that it was their responsibility to guide and support GBMSM in a non-judgmental manner.

#### Require justification

The engagement activities aimed at making religious leaders understand and justify their own opinions and beliefs toward GBMSM within the context of HIV and religion. This was done through group exercises that asked the religious leaders to provide rationale for their prejudices. For instance, religious leaders would prepare a joint sermon on stigma and discrimination using only the messages from the Bible and Quran they would have used before the training. They were then required to role-play and imagine that they were GBMSM members or were living with HIV seated through the sermon. In the same example, religious leaders were asked, guided by questions, to reflect on the tone of their sermon, was it inviting, compassionate and loving or would they feel the sermon was scary and condemning? Would GBMSM be willing to come back to the church or mosque?

#### Foster researcher relationships

Throughout the sessions, emphasis was placed on calm, respectful attitude and behavior toward each other regardless of differences in opinion. Substantial time was assigned to making the religious leaders feel valued throughout the discussions about the importance of their work. When religious leaders reflected on ethical implications of working with GBMSM members, they mentioned fear to receiving negative treatment from their religious superiors if interacting with this legally defined unaccepted population. With time, however, through continued engagements, religious leaders tended to feel they could freely interact with the GBMSM members of the research team, and express themselves in authentic, relational and self-sharing ways. With encouragement, leaders ascertained that they could sit together, and have a conversation with GBMSM (research) members. This was a situation that before the facilitated engagements they would have resisted. Consensus to collaborate with GBMSM researchers was a concrete step that linked the religious leaders psychologically to the GBMSM members, which can be key to sustaining reduced prejudicial attitudes on a long term.

Two significant positive outcomes materialized after the engagement sessions: a long-term commitment to meetings between religious leaders and the HIV researchers, and the initiation of a ‘transformational working group’ among the religious leaders themselves to build a strong, united framework that respects individual, community, religious, and cultural diversity, which at the same time focuses on healing GBMSM hurts and challenges mistrust in their communities.

However, social, emotional and spiritual changes are large and complex issues and take time. Although the involvement and commitment of sensitized religious leaders seemed to hold promise for having a powerful impact on ensuring continuous access to HIV care for GBMSM on the coast of Kenya, there were events that we were told about that give us a reason to think that more work is needed. For example, one of the engaged and sensitized religious leaders, who had previously been involved in the homophobic attack at the HIV research clinic, reverted to his ‘old’ way of talking about GBMSM while addressing a different forum. Another relapse occurred, when one of the positively changed religious leaders was – for some time – ex-communicated by his superiors for having taken part in the engagement activities.

Realizing that lack of endorsement by religious leaders’ superiors could limit the effect of community engagements’ intention to reduce discrimination against GBMSM, a more holistic approach was then needed. The research team started individual sessions with the religious leaders involved, facilitating personal reflective internal conversation, while the superior, non-sensitized religious leaders, responsible for ex-communication of their colleague, were invited for collective exploration and solution finding. Both the engagements resulted in the same insight deriving from scripture: ‘to do unto others as you would do unto yourself’, and a concluding variant of the rule: ‘to do unto yourself as you would do unto others’.

## Discussion

Across the African continent, e.g. in Ethiopia (Endeshaw et al., 2017), Mozambique (Agadjanian & Menjivar, 2011), Nigeria (Ezeanolue et al., 2015), and Tanzania (Watt et al., 2009), responses to the HIV epidemic have involved religious leaders but their support to the HIV prevention and care efforts have been predominantly convergent with religious programs, in form and content*.* In Kenya, churches and mosques remain conservative in their values about sexuality, and as a result create obstacles to airing social and health problems caused by homophobia, heterosexism, and HIV stigma (Mbote et al., 2016).

Engaging religious leaders from the coast of Kenya resulted in a positive dialog between religious leaders, GBMSM members and HIV researchers. Reflection exercises aided religious leaders to become aware of their own stigmatizing actions and helped transform their habitual use of discriminatory language. Religious leaders also showed that they were able to gradually apply more humanistic, caring discourse, indicating that one can interrupt the cycle of socialization and stand up for change (Harro, 2000). This was possible by supporting religious leaders in their understanding of sexuality and reflection, rather than criticizing or excluding them, or undermining their importance in their respective communities. The reflexive process was instrumental in offering insights to advancing knowledge, and developed a space in which the majority of the group could come to terms with conflicts between religion and recognizing GBMSM existence. Through the engagement sessions the religious leaders became more aware of the activities of the research clinic and felt included and appreciated, rather than left out.

In line with several other studies that have shown that working closely and collaboratively with religious leaders is successful for HIV prevention and care (Duff & Buckingham, 2015; Obong’o, Pichon, Powell, & Williams, 2016; Roman Isler, Eng, Maman, Adimora, & Weiner, 2014; Szaflarski et al., 2013; Taegtmeyer et al., 2013; Williams, Palar, & Derose, 2011; Willms et al., 2011), engagement interventions presented in this paper indicate that when religious leaders reflexively (re)position themselves, changes in views regarding homosexuality can emerge. Our findings, therefore, contribute to existing literature by demonstrating that on-going engagement processes, respectful work, and collaboration in an openly violent environment can generate a more accepting discourse of GBMSM. Given the hostility against same-sex relationships in much of Africa, collaborations with religious leaders are vital, or results can be detrimental as the attack to research clinic suggests.

In Kenya, importance of collaborations with religious leaders is underlined by the fact that the Kenyan state does not recognize the rights of GBMSM. South Africa is the only country in Africa that has decriminalized same-sex relations. Burchardt has shown how rights against sexual and racial discrimination and reasoned secularity have become part of liberal values of individual freedoms and human rights in South Africa (Burchardt, 2015). In Brazil, Muñoz-Laboy and colleagues have shown that historically Brazil’s state-defined non-discrimination policies, and closely related national HIV response being channeled through religious organizations, seems to have enhanced GBMSM access to HIV prevention and health care (Munoz-Laboy, Garcia, Moon-Howard, Wilson, & Parker, 2011; Murray, Garcia, Munoz-Laboy, & Parker, 2011). In contrast, in many African countries where decriminalization of homosexuality may be difficult or even impossible in the near future, involvement of, engagement with, and support from religious leaders is essential for creating supportive spaces and access to health care for GBMSM, which, as we have shown, can also enhance community tolerance.

While the results are positive, it is necessary to acknowledge their limitations. First, the sample size was relatively small and not representative of religious leaders elsewhere in the country. Second, the engagement intervention was not planned as a randomized, controlled intervention and thus religious leaders’ attitudes were not measured in a structured way. Third and most importantly, the engagement activities cannot claim success in changing every leader in every aspect of their prejudices towards GBMSM. While participants in the process seemed genuinely moved and touched by the process, it is not possible to rule out some social desirability bias in their reported experiences during the engagement. A further study will be required to establish the effect of the engagement intervention on religious leaders’ long-term attitudes towards GBMSM, as well as what practical contribution religious leaders may make to support HIV prevention and care for GBMSM.

## Conclusion

This study has demonstrated that collaboration between religious leaders, GBMSM members and researchers can achieve notable shifts in knowledge, views and discourses towards GBMSM across a range of religious leaders. The carefully structured participatory processes gave opportunities for religious leaders to interrogate and justify the bases of their perspectives, to reflect on and feel the effects of their attitudes towards GBMSM, and allowed them to engage on an emotional level with GBMSM. The following recommendations can possibly serve as potentially effective strategies: (1) improve religious leaders’ understanding of why GBMSM are targeted for HIV prevention and care; knowledge was particularly empowering for religious leaders as they indicated that if they changed their attitude towards GBMSM, community members would follow their example. (2) Have religious leaders interact with GBMSM to improve mutual understanding and reduce anxiety about interacting with GBMSM. (3) As the religious leaders recognized their authority in mitigating tensions in their respective communities, explain GBMSM research, especially when new initiatives are taken; and (4) Stress religious leaders’ central position in the community with the opportunity to enhance GBMSM’s access to HIV prevention and care; religious leaders realized that they have a role to play in facilitating GBMSM social acceptance to advance their access to HIV prevention and care. Finally, the engagements with religious leaders in Malindi demonstrated that religious leaders are willing to articulate their judgments differently, given the right support and motivation to do so.

## Disclosure statement

No potential conflict of interest was reported by the authors.

## Funding

This work was supported by the US Agency for International Development (USAID). Work with key populations in Kilifi, Kenya is funded by the International AIDS Vaccine Initiative. IAVI’s work is made possible by generous support from many donors including: The Bill & Melinda Gates Foundation; the Ministry of Foreign Affairs of Denmark; Irish Aid; the Ministry of Finance of Japan; the Ministry of Foreign Affairs of the Netherlands; the Norwegian Agency for Development Cooperation (NORAD); the United Kingdom Department for International Development (DFID), and the United States Agency for International Development (USAID). The full list of IAVI donors is available at www.iavi.org. The KWTRP at the Centre for Geographical Medicine Research-Kilifi is supported by core funding from the Wellcome Trust (grant #077092). The contents are the responsibility of the study authors and do not necessarily reflect the views of USAID, the US government or the Wellcome Trust.
